# A Sustainable Approach: Cellulose-Based Biodegradable Water-Retaining Agents Facilitating Mine Restoration in Arid Xinjiang Mining Zones

**DOI:** 10.3390/polym18212670

**Published:** 2026-10-28

**Authors:** Kaigang Li, Naeem Akram, Lei Zhou, Zhanwen Cao, Huishan Zhang, Ali Ahmad, Zhenning Cheng, Aizong Wu, Wenlong Mo, Jia Guo

**Affiliations:** 1 School of Chemical Engineering, Xinjiang University, Urumqi 830017, China; likaigang@stu.xju.edu.cn (K.L.); aizongwu@126.com (A.W.); mowenlong@xju.edu.cn (W.M.); 2 Xinjiang Energy Co., Ltd., Urumqi 830026, China; 13919372459@139.com (Z.C.); 13999976521@139.com (H.Z.); hmsthczn@163.com (Z.C.); 3 School of Chemical Engineering, Minhaj University Lahore, Lahore 54000, Pakistan; engrali@sdut.edu.cn; 4 Administration Committee of Chemical Industry Park, Midong District, Urumqi 831400, China; 18199134666@163.com; 5 School of Mechanical Engineering, Shandong University of Technology, Zibo 255000, China

**Keywords:** biodegradable water-retaining agents, cellulose, mine ecological restoration, saline-alkali soil remediation, non-soil replacement restoration

## Abstract

**Highlights:**

**What are the main findings?**

- A biodegradable cellulose-based WRA (CCM−CMC) was synthesized.
- The prepared WRA exhibits high swelling capacity as an eco-friendly option.
- Its application enhanced soil water retention by up to 39.2%.
- It markedly improved plant growth and achieved over 80% survival rate.

**What are the implications of the main findings?**

- This material enables the restoration of saline-alkali mine land without topsoil replacement.

**Abstract:**

To address the poor biodegradability and high environmental risks of traditional synthetic water-retaining agents (WRAs), and the vegetation colonization challenge induced by organic matter deficiency in saline-alkali mining areas, a biodegradable WRA (CCM−CMC) was facilely synthesized from natural cellulose (CCM) and carboxymethyl cellulose (CMC) with epichlorohydrin (ECH) as a crosslinking agent. An in situ non-topsoil replacement remediation system of “superabsorbent polymer–biological fertilizer–native stress-tolerant plants” was established, and its water absorption-retention performance, biosafety, and ecological remediation efficacy were systematically evaluated. CCM−CMC displayed excellent equilibrium swelling ratios (1218, 443, 305, 148 g/g in distilled water, tap water, mining reclaimed water, and 0.9 wt% NaCl solution, respectively), with a 57.28% weight loss after 30-day soil burial, verifying favorable biodegradability. Soil application of CCM−CMC improved soil water-holding capacity by up to 39.20%, and maximally increased *pakchoi* germination rate, plant height, and dry weight by 23.33%, 327.4%, and 430.8% under drought stress. Field trials in Xinjiang arid mining areas achieved an over 80% survival rate for over ten native stress-tolerant plants, and significantly ameliorated the soil physicochemical properties. These eco-friendly WRAs and derived remediation technology avoid the high costs and ecological disturbance of topsoil replacement, providing a practical technical strategy for the ecological restoration of arid and highly saline-alkali mining areas.

## 1. Introduction

Xinjiang is abundant in coal and other mineral resources, which serve as an important pillar of regional economic growth. Xinjiang lies deep within the interior of the Eurasian continent and experiences a typical arid to semi-arid climate [1]. Its annual average precipitation is far lower than the evaporation capacity, coupled with severe water scarcity and an extremely fragile ecological environment. In addition, the problem of serious soil salinization has made ecological restoration in mining areas face enormous challenges.

Water-retaining agents (WRAs) are typically linear hydrophilic polymers cross-linked either chemically or physically. Rich in a large number of hydrophilic groups, they demonstrate the capacity to absorb as well as retain substantial amounts of water [2,3], thus holding broad prospects for addressing water scarcity issues in crop cultivation in arid regions [4,5,6]. Three major categories of WRAs are recognized based on their synthetic raw material differences [7]. The synthetic category includes polyacrylic acid, polyacrylamide, and polyacrylonitrile [8]; the semi-synthetic category includes starch-polyacrylamide and natural polymer-polyacrylic acid [9]; and the natural category includes cellulose and chitosan [10]. At present, most commercially available WRA products fall into the synthetic category, which are not only high in cost but also poor in degradability; moreover, they may cause problems such as secondary soil pollution, thereby limiting their applications in agriculture and ecological restoration [11]. In contrast, natural polymer-based WRAs, especially polysaccharide-based WRAs, have attracted considerable attention from researchers owing to their superior characteristics, including biocompatibility, biodegradability, renewability, and non-toxicity [12,13,14].

Although numerous studies have demonstrated that polysaccharides such as starch [15,16], sodium alginate [17], chitosan [18], chitin, cellulose [19] and its derivatives [20], lignin, carrageenan, xanthan gum, pectin, and guar gum [21] can serve as raw materials for synthesizing biodegradable WRAs, several challenges still restrict their large-scale application. Wu et al. synthesized a water-retaining agent based on carboxymethyl cellulose and polyaspartic acid, which exhibited swelling capacities of 60 g/g in deionized water and 50 g/g in 0.1 mol/L NaCl solution. This material contributes to agricultural water conservation [20]. First, most biodegradable water-retaining agents exhibit poor swelling properties, and their water absorption and retention efficacy are susceptible to inhibition by metal ions and saline-alkali conditions, which compromise their overall functionality [22]. Second, they possess inferior mechanical properties. Under mechanical disturbance in complex terrain, soil wetting–drying cycles, and high-salinity environments, they are prone to structural disintegration or uncontrolled swelling, resulting in insufficient environmental stability and a shortened water and fertilizer retention period. Third, high-performance water-retaining agents usually have relatively high production costs. Hence, developing water-retaining agents featuring high swellability, mechanical robustness and biodegradability via low-cost raw materials and synthetic strategies remains a major challenge [23].

Cellulose (CCM), which ranks as the most abundant natural polymer globally, possesses characteristics such as biodegradability, non-toxicity, and renewability [24]. The hydroxyl groups of cellulose endow the material with a certain water absorption capacity, while the excellent mechanical strength and thermal stability of its crystalline regions provide skeletal support for the polymer network structure [25]. Carboxymethyl cellulose (CMC) is an anionic derivative obtained by the carboxymethylation modification of cellulose [26]. The introduction of carboxymethyl groups destroys the crystalline structure of cellulose, rendering CMC soluble in both cold and hot water and thus facilitating the operation of synthetic reactions. Furthermore, the carboxyl groups (-COOH) are a strongly hydrophilic moiety that also serves as a crosslinking site. Compared to cellulose’s hydroxyl groups, it exhibits higher reaction selectivity and efficiency. The abundance of hydroxyl and carboxyl groups endows the polymer network with excellent water absorption properties [27,28]. CCM and CMC are widely available and stably supplied, significantly reducing the preparation cost of WRAs. Both are biodegradable materials, and the resulting water-retaining agents can be completely degraded by microorganisms in natural environments (e.g., soil and water bodies), ensuring high biosafety. The application of cellulose and carboxymethyl cellulose conforms to the development concept of greening and functionalization for modern materials [29,30].

This study aims to develop a natural polymer-based superabsorbent polymer with both excellent swelling performance and biodegradability, which is suitable for arid and highly saline-alkali environments, via a simple and efficient preparation process. Specifically, the CCM−CMC superabsorbent polymer was synthesized using CMC and CCM as raw materials via crosslinking with epichlorohydrin (ECH). After optimizing the synthesis conditions via single-factor experiments, the morphology, structure, and thermal stability were characterized using techniques such as scanning electron microscopy (SEM) and Fourier transform infrared spectroscopy (FTIR); the material’s swelling characteristics, biocompatibility and safety under different environmental conditions were systematically evaluated; and the soil improvement effect and crop growth promotion potential of CCM−CMC under drought stress were verified via soil water retention tests and drought stress cultivation simulation of pakchoi. Targeting the habitat characteristics of severe cold climate, highly saline-alkali soil and low organic matter content in arid mining areas of Xinjiang, as well as the practical bottlenecks of high cost and poor adaptability associated with traditional topsoil replacement remediation technology, this study further conducts field trials on cultivating drought-tolerant plants in mining areas, systematically clarifies the field application efficacy of the CCM−CMC WRAs, and thus intends to provide a scientific and cost-effective technical support scheme for the ecological restoration of arid and highly saline-alkali mining areas.

## 2. Materials and Methods

### 2.1. Materials

Carboxymethyl cellulose (CMC) with a degree of substitution of 0.7 and a viscosity of 200–500 mPa·s, α-cellulose (CCM, 50 μm particle size), and (±)-epichlorohydrin (ECH, ≥99.5%) were supplied by Aladdin Biochemical Technology Co., Ltd. (Shanghai, China). Urea and sodium hydroxide (NaOH) came from Innochem Technology Co., Ltd. (Beijing, China). Additional reagents: sodium chloride (NaCl), calcium chloride (CaCl_2_), ferric chloride (FeCl_3_), sodium carbonate (Na_2_CO_3_), and sodium sulfate (Na_2_SO_4_) were also obtained from Aladdin. All chemicals and solvents were of analytical grade, purchased from Aladdin Biochemical Technology Co., Ltd., Shanghai, China, and used without further purification.

### 2.2. Preparation of CCM−CMC Water-Retaining Agents

Three grams of CCM was added into 100 mL mixed solvent consisting of 6 wt% NaOH and 4 wt% urea, followed by stirring at room temperature to form a white suspension. The suspension was frozen in a refrigerator for 24 h and then thawed under stirring at room temperature to obtain a cellulose solution with a certain concentration. Separately, 3 g of CMC was dissolved in the 6 wt% NaOH/4 wt% urea solution under stirring at room temperature.

After mixing the previously prepared CCM and CMC solutions, epichlorohydrin (ECH) was added while stirring at ambient temperature. The homogeneous mixture was then heated in a thermostatic water bath at a preset temperature to allow crosslinking. Once the reaction finished, the product was rinsed repeatedly with distilled water to remove residual solvent, unreacted monomers, and leftover crosslinker. Finally, it was dried and ground into the finished product.

A series of CCM−CMC WRA samples were obtained by varying the mixing ratio of CCM solution to CMC solution and the dosage of ECH.

### 2.3. Characterization of CCM−CMC

The structure, morphology, physicochemical properties, and thermal stability of the CCM−CMC WRAs were characterized.

A Thermo Fisher Nicolet Is5 spectrometer (Thermo Fisher Scientific, Madison, WI, USA) was used to collect Fourier transform infrared (FTIR) spectra. The powder samples were ground with KBr, compressed into pellets, and then analyzed by infrared spectroscopy. Spectra were recorded over a wavenumber range of 4000–400 cm^−1^ at a resolution of 4 cm^−1^.

A Malvern Panalytical X’Pert^3^ Powder X-ray diffractometer (Malvern Panalytical, Almelo, Netherlands) was used to obtain the XRD patterns of the samples. The measurements were performed at a scan rate of 2°/min over a 2θ range of 5 to 90°.

A thermogravimetric analyzer (Mettler Toledo TGA/DSC 3+, Mettler Toledo, Greifensee, Switzerland) was used to evaluate the thermal stability of the samples. Measurements were carried out at a heating rate of 10 °C/min over a temperature range of 30–600 °C.

Scanning electron microscopy (SEM, Thermo Fisher, Quattro S) was utilized to characterize the surface morphology of WRAs samples. Before the test, the freeze-dried WRAs samples were subjected to brittle fracture and gold-coated under vacuum.

The wettability of the WRA samples was tested using a contact angle meter (Kruss, DSA30S, KRÜSS GmbH, Hamburg, Germany), with deionized water as the liquid medium.

### 2.4. Mechanical Properties Determination

An electronic universal testing machine (WANCE TSE104B, WANCE Testing Machine Co., Ltd., Jinan, China) was used to evaluate both compressive and tensile properties of the fresh CCM−CMC WRAs. For compression tests, cylindrical samples (height 10 mm, diameter 15 mm) were prepared, and stress–strain curves were acquired at 10 mm/min. Tensile tests were performed on strip specimens (25 mm × 10 mm × 5 mm) under the same loading rate of 10 mm/min [20].

### 2.5. Swelling Behavior & Kinetic Modeling in Various Media

The swelling properties of the WRAs were determined using the weighing method in distilled water, tap water, reclaimed water, and physiological saline, respectively [7]. The mass of the dried WRA was accurately weighed and recorded as *M*_1_, and then the WRA was placed into a tea bag of known mass (the mass of the tea bag was recorded as *M*_2_). Subsequently, at room temperature, the tea bags loaded with SAP were immersed in different types of liquids, respectively. At predetermined swelling time points, the combined mass of the swollen WRA and the tea bag was measured and recorded as *M_3_*. The swelling ratio (*Q*) at different time points was calculated according to Equation (1). Among the liquids, distilled water was laboratory-prepared, tap water was collected from the laboratory tap water supply, reclaimed water was the re-treated domestic wastewater from arid mining areas in Xinjiang, and physiological saline was the 0.9 wt% NaCl solution prepared in the laboratory.

(1) $$Q=\frac{M_{3}-M_{1}-M_{2}}{M_{1}}$$

To gain deeper insight into the water absorption kinetics of the WRA, the experimental data were fitted using both pseudo-first-order and pseudo-second-order kinetic models. The equations corresponding to these two models were shown in Equations (2) and (3), respectively.

(2) $$\ln({Q_{e}-Q}_{t})=\ln Q_{e}-k_{1}t$$

(3) $$\frac{t}{Q_{t}}=\frac{t}{Q_{e}}+\frac{1}{k_{2}Qe^{2}}$$

Here, *Q_e_* stands for the swelling rate of the WRA at equilibrium, *Q_t_* refers to the swelling rate measured at time *t*, while *k*_1_ and *k*_2_ are the respective rate constants associated with the pseudo-first-order and pseudo-second-order kinetics [20].

### 2.6. Repetitive Water Absorption Performance Measurement

A total of 0.1 g of dried CCM−CMC WRA was weighed and transferred into a tea bag. The tea bag containing the WRA was placed in distilled water and allowed to swell until equilibrium was reached. The swelling ratio (*Q*) was then determined using Equation (1). Afterward, the swollen material was dried in an air blast drying oven at 40 °C. This swelling–drying cycle was repeated five times.

### 2.7. Water Retention Properties at Different Temperatures

A total of 0.1 g of dried CCM−CMC WRA was weighed and transferred into a tea bag. The WRA-loaded tea bag was immersed in distilled water until swelling equilibrium was reached, then placed in an air blast drying oven at different temperatures for dehydration. The weight of the sample was measured at different drying time intervals, and the swelling ratio (*Q*) of the WRA was calculated according to Equation (1).

### 2.8. Measurement of Soil Water-Holding Capacity (SWC) and Evaporation Rate

One kg of ordinary soil was thoroughly mixed with 0.25 g (0.025 wt%), 0.50 g (0.05 wt%), and 0.75 g (0.075 wt%) of dried WRA, respectively. The mixtures were placed into plastic flowerpots of uniform specifications with holes at the bottom. The flowerpots were then set in trays filled with tap water, allowing the soil to reach a fully saturated state within 24 h via capillary rise. Subsequently, the trays were removed, and the flowerpots were drained of any excess free water present at their base. The flowerpots were weighed to obtain the maximum water-holding capacity of the soil. In the control group, the dosage of SAP was set to 0 g.

The saturated soil-filled flowerpots were transferred into an air blast drying oven at 30 °C for the water evaporation test. The weight changes of the flowerpots were recorded at different time points until the weight stabilized. Based on the difference between the weighed mass at each time point and the total mass at maximum water-holding capacity, the temporal water evaporation loss of the soil was calculated.

### 2.9. The Biodegradability Measurement

The soil burial method was employed to evaluate the biodegradability of the CCM−CMC WRA. Seven groups of dried CCM−CMC samples (1 g per group) were separately placed into tea bags (PP non-woven fabric) and immersed in distilled water until reaching swelling equilibrium. Swollen CCM−CMC samples enclosed in tea bags were buried at a depth of 5 cm beneath the soil surface (the soil was collected from arid mining areas in Xinjiang, and the soil moisture was maintained at 60% of the maximum soil water-holding capacity). The soil containers were incubated at 25 °C for 30 days. On days 1, 5, 10, 15, 20, 25, and 30, one group of tea bags was carefully retrieved. The samples were gently rinsed with absolute ethanol to remove any adhering soil particles, then dried to a constant weight and weighed for the record [7]. The percentage of biodegradation rates was calculated using the following formula:

(4) $$Biodegradation\;rates\;\left(\%\right)\;=\;(1\;-\;\frac{W_{0}\;{-\;W}_{t}}{W_{0}})\;\times \;100\;$$

where *W*_0_ is the initial dry weight of the hydrogel sample before biodegradation, and *W_t_* is the residual dry weight of the hydrogel after degradation for a specified period.

### 2.10. Cytotoxicity Assay

The safety of the superabsorbent polymer (SAP) product could be reflected by determining the effects of SAP samples with different concentrations on cell viability. The CCK-8 assay was employed to detect both cell proliferation and cytotoxicity. A microplate reader was used to measure the optical density (OD) at 450 nm, which indirectly reflects the quantity of viable cells present.

Normal human dermal fibroblasts (NHDFs) were used for the CCK-8 cytotoxicity assay. The SAP samples were subjected to ultraviolet (UV) sterilization. After sterilization, the concentrations of the samples were adjusted to 50 mg/mL, 25 mg/mL, 12.5 mg/mL, 6.25 mg/mL, and 3.13 mg/mL, and the samples at various concentrations were added to the culture system for co-culture with cells. Three groups were set up: the experimental group (cells + medium + WRAs), the control group (cells + medium + no WRAs), and the blank control group (medium + no WRAs). The microplates were placed in a cell incubator for further incubation for 24 h prior to detection.

### 2.11. Animal Dermal Contact Toxicity Assay

The dermal contact safety of the WRAs could be evaluated via the single dermal sensitization test in mice. The hair on the dorsal skin area of each mouse (2 cm × 3 cm) was shaved, and the residual hair stubble was removed with depilatory cream. Subsequently, the swollen SAP was evenly applied to this skin area, which was then covered with two layers of gauze and secured using non-irritating medical bandages to prevent detachment of the test substance and ingestion by the mice. After 4 h of application, the residual samples were rinsed off with water. The systemic toxic manifestations and mortality of the mice were observed for three consecutive days. The skin reactions were scored for erythema and edema according to Supplementary Materials, Table S1, and a comprehensive evaluation was performed based on the mean score of the mice. The irritation intensity was determined according to Supplementary Materials, Table S2, using the mean value of the highest scores recorded at each time point. A physiological saline contact control group was set up, with each group repeated three times. The mice used in the control group and the experimental group were of the same sex, consisting of two male mice and one female mouse, respectively (*n* = 3). All procedures involving animals were conducted in accordance with the ARRIVE guidelines (Animal Research: Reporting of In Vivo Experiments).

### 2.12. Study on Simulated Cultivation of Pakchoi Under Drought Stress and the Regulatory Effect of CCM−CMC

Plastic flowerpots, each containing 1 kg of soil, were supplemented with WRAs at mass fractions of 0, 0.025%, 0.05%, and 0.075%, respectively. Thirty *pakchoi* seeds were sown per flowerpot, with three replicates set for each treatment. During the initial planting stage, the soil moisture in the flowerpots was maintained at 80% of the maximum water-holding capacity to facilitate seed germination. After seed germination was completed, seedling thinning was performed, and 5 robust seedlings with uniform growth were retained per flowerpot. Subsequently, the soil water-holding capacity of each group was regulated to 40%, 50%, and 60%, so as to simulate the growth environments under severe drought, moderate drought, and mild drought conditions, respectively. Throughout the experiment, water was replenished every two days to maintain the preset soil water-holding capacity. When the pakchoi seedlings developed 6 leaves, morphological indices, including root length and plant height, were measured.

### 2.13. Field Experiments for Ecological Restoration in Mining Areas

A dedicated experimental area was designated in an arid mining area of Xinjiang to conduct the field application test of CCM−CMC [31]. The experiment adopted the drip irrigation method, with the irrigation water being the treated domestic wastewater from the mining area that met the discharge standards. The application dosage of CCM−CMC WRA was set at 1 kg/mu, and that of the biological fertilizer was 130 kg/mu. Subsequently, drought- and salt-tolerant plants, including *Suaeda glauca*, *Ammopiptanthus mongolicus*, *Hedysarum scoparium*, and *Sacsaoul* were planted in the experimental area [32]. By systematically determining relevant parameters such as plant survival rate, soil pH value, total salt content, and soil organic matter content, the practical application effect of CCM−CMC under the conditions of arid, frigid, saline-alkali soil in mining areas was evaluated.

### 2.14. Statistical Analysis

All quantitative experimental data were calculated as the average of three parallel experiments.

## 3. Results and Discussion

### 3.1. Effect of Different Reaction Conditions on the Swelling Properties of WRAs

**Figure 1:**
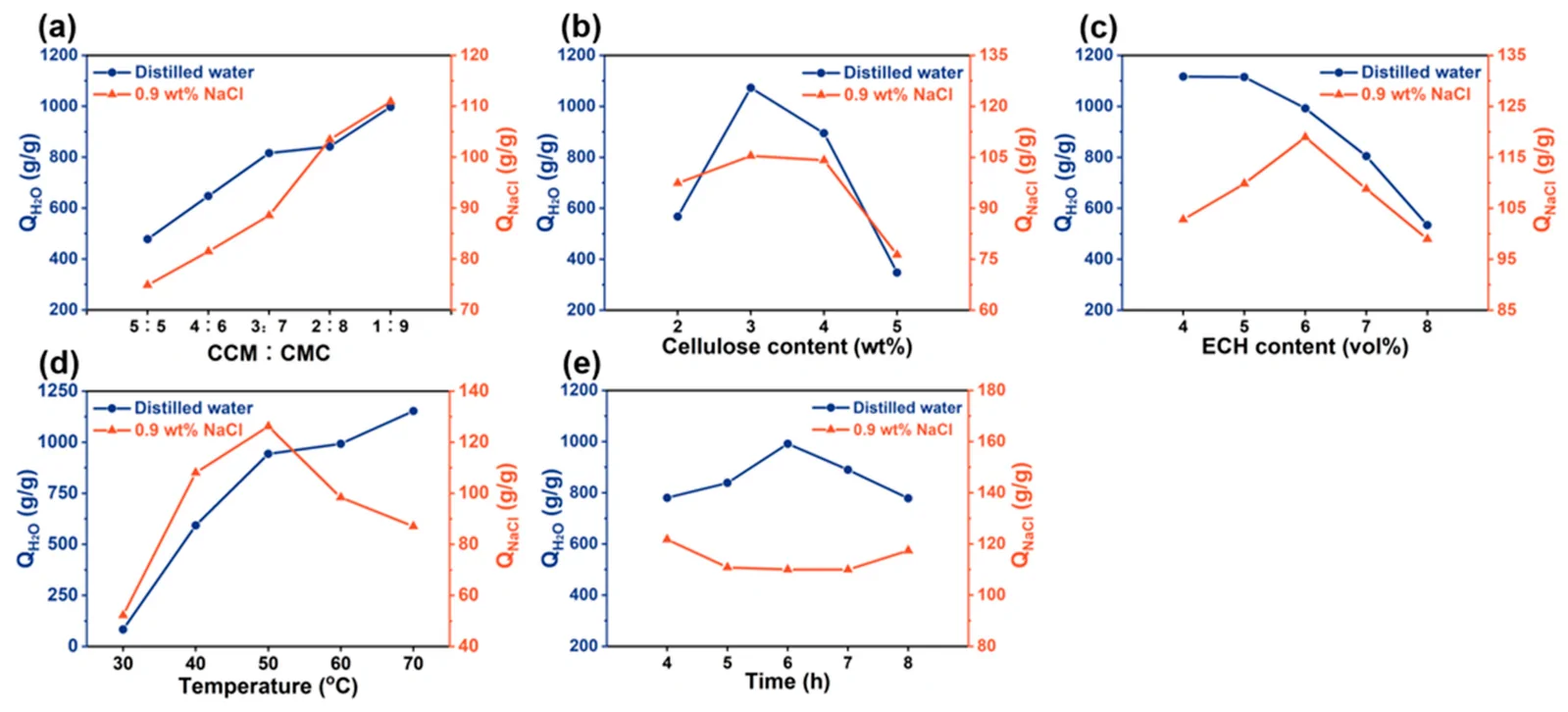
Effect of Different Conditions on the Swelling Performance of WRAs. (**a**) Monomer ratio, (**b**) cellulose concentration, (**c**) the dosage of crosslinking agent ECH, (**d**) reaction time, and (**e**) reaction temperature.

The impact of various reaction conditions on WRA behavior was studied, and the outcomes are displayed in Figure 1. The hydrophilic groups contained in the WRA network directly affect the formation of hydrogen bonds between the polymer and water molecules, thus exerting a remarkable influence on its swelling characteristics. As shown in Figure 1a, the swelling performance of the WRAs is significantly enhanced with the increase in CMC content. This is because CMC is rich in hydroxyl and carboxyl groups as hydrophilic moieties; the hydroxyl groups enlarge the distance between molecular chains, which further contributes to the increased water and salt absorption capacities of WRAs. When the mass ratio of CCM to CMC is 1:9, the swelling ratio of WRAs in distilled water reaches 996.65 g/g. With a further rise in CMC content, the synthesized WRAs present a semi-dissolved state in water. This phenomenon arises from the skeletal support effect of cellulose in the WRA matrix: an insufficient cellulose content cannot form a stable three-dimensional network structure of the WRA.

As shown in Figure 1b, the swelling capacity of the WRAs first increases and then decreases with increasing cellulose content. When the mass fraction of cellulose is below 2 wt%, the insufficient cellulose content fails to provide an effective skeletal support, resulting in unstable swelling behavior of the prepared WRAs. As the cellulose mass fraction increases, its solubility gradually decreases. When the mass fraction of cellulose exceeds 5 wt%, the cellulose cannot be completely dissolved.

Figure 1c shows how the performance of the WRAs varies with the amount of cross-linking agent used. As the ECH increases, the swelling rate of the WRAs in distilled water decreases significantly. This is because, under the condition that an effective three-dimensional polymer network structure can be formed, a further increase in ECH content enhances the crosslinking degree, resulting in tighter crosslinks and reduced pore sizes within the network, thereby impairing the swelling performance. However, the swelling rate of the WRAs in a 0.9 wt% NaCl solution first increases and then decreases with increasing crosslinker content. This may be attributed to the influence of microporous size formed at a certain crosslinking degree on cationic swelling. When the ECH content was 6 vol%, the swelling ratio in 0.9 wt% NaCl solution reached a relatively high value.

The performance of the water-retaining agent as functions of reaction temperature and reaction time are presented in Figure 1d,e, respectively. A gradual rise in reaction temperature improves monomer crosslinking efficiency and increases the crosslinking degree of the polymer network, enhancing swelling performance in distilled water. Further temperature elevation accelerates polymerization. The resultant high polymerization rate decreases the average relative molecular mass of the polymer and impairs swelling capacity in 0.9 wt% NaCl solution. In addition, swelling performance rises first and then falls with increasing reaction time. A short reaction time yields low crosslinking degree and inferior swelling behavior. As the reaction continues, unreacted ECH reacts with monomers to build a crosslinked network. However, excessive crosslinking suppresses swelling performance. Notably, reaction time has little effect on the swelling property in salt solution.

As crosslinking efficiency is sensitive to varying reaction conditions, the yield of CCM−CMC (calculated from the masses of CMC and CCM) was in the range of 70–90% in the single-factor experiments. When CCM:CMC = 1:9, the CMC concentration was 3 wt%, ECH loading was 6 vol%, reaction duration was 6 h, reaction temperature was 60 °C, and the average yield of CCM−CMC reached above 85%.

### 3.2. Performance Test and Reaction Mechanism Investigation of CCM−CMC

#### 3.2.1. Morphological Characteristics of CCM−CMC

On the basis of the observed effects that distinct reaction conditions have on the swelling characteristics of the WRAs, the optimized preparation parameters were determined as follows: the mass ratio of CCM to CMC was 1:9, the dosage of ECH was 6 vol%, and the reaction was carried out at 50 °C for 6 h to prepare a WRA sample (CCM−CMC) with excellent performance. The hydrophilicity, mechanical properties, and micromorphology of this sample were tested, and the reaction mechanism was further explored.

**Figure 2:**
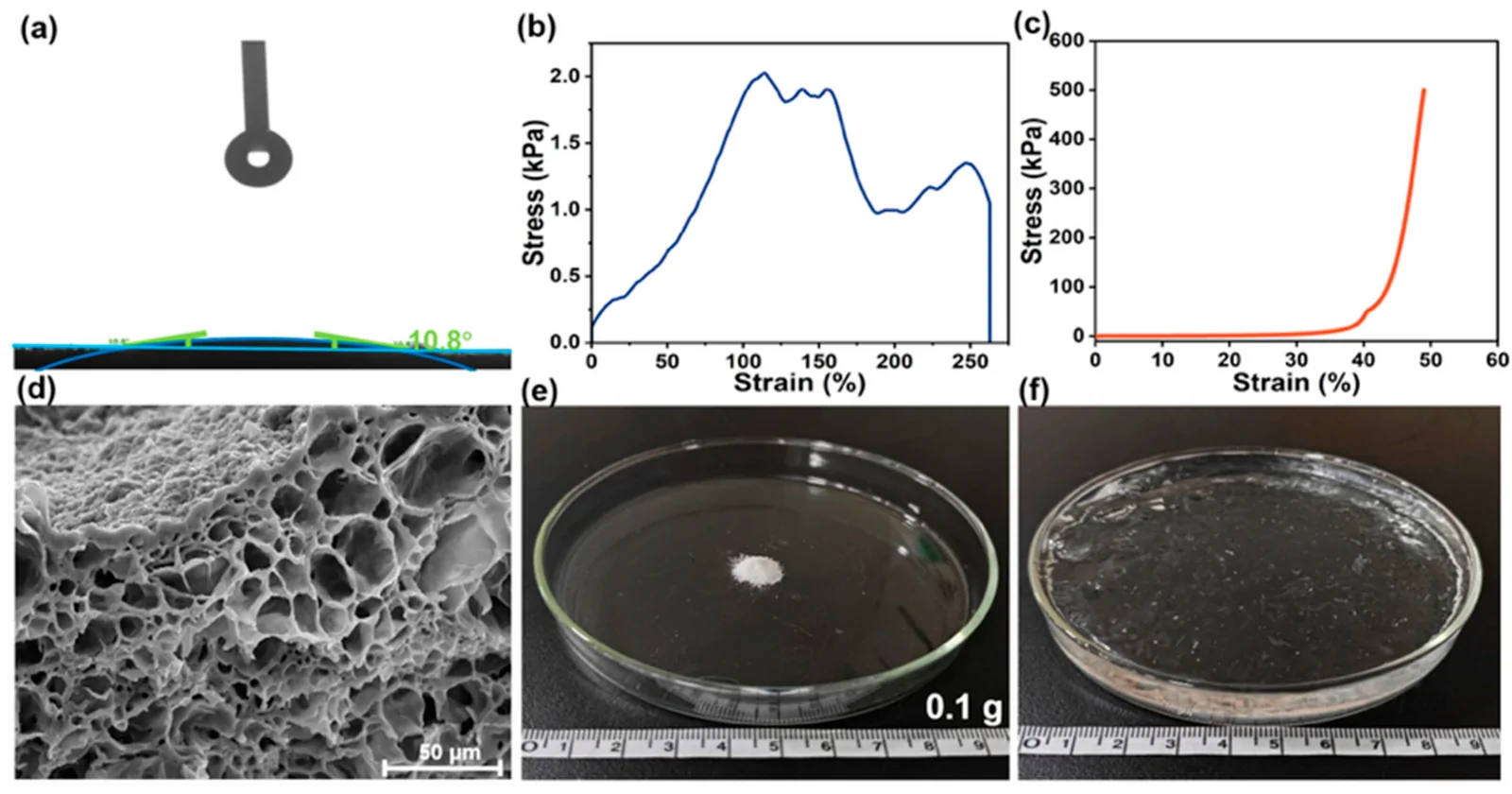
Morphological characteristics of CCM−CMC. (**a**) contact angle, (**b**) tensile property test, (**c**) compression property test, (**d**) SEM image, (**e**) dry state, and (**f**) CCM−CMC after swelling in distilled water.

The contact angle measurement results of CCM−CMC are presented in Figure 2a. Deionized water spread rapidly and instantaneously on the surface of CCM−CMC, with a contact angle of 10.8° (<<90°), which indicates that CCM−CMC possesses excellent hydrophilicity. Moreover, a smaller contact angle corresponds to a greater interaction strength between the superabsorbent polymer and water at the soil–particle interface, leading to better water adsorption and increased soil water-holding capacity.

Figure 2b displays the mechanical properties of CCM−CMC. The material has low tensile strength yet good tensile elongation, with a tensile elongation of 260% and a maximum tensile stress of 2 kPa. Upon stretching, stress shows a fluctuating trend, increasing initially and then decreasing with growing strain. This phenomenon may originate from the yielding behavior of the polymer. At a specific strain, polymer molecular segments rearrange to adapt to deformation. Molecular chain slippage sustains the applied strain and causes progressive cracking of fiber bundles, resulting in the fluctuating stress–strain response. The compression-fracture results in Figure 2c reveal the favorable compression resistance of CCM−CMC. A compressive stress of 500 kPa is obtained at 50% compression, demonstrating that the material has sufficient compressive strength when applied in soil and can reduce the risk of structural collapse under soil pressure.

Figure 2d displays the SEM images of CCM−CMC. As shown in the figure, the superabsorbent polymer still had a multi-level interconnected porous structure at the scale of 50 μm, with uniform and abundant pore distribution. This porous structure not only endows the superabsorbent polymer with excellent swelling performance, but also provides a certain structural support for the material. The dry and swollen states of CCM−CMC are shown in Figure 2e,f, respectively. The freshly prepared superabsorbent polymer was a transparent elastomer, which turned into a white solid with significant volume shrinkage after drying and was ground into a white powder. The specific surface area of the superabsorbent polymer was increased after grinding treatment, which significantly improved its swelling rate.

#### 3.2.2. Investigation on the Reaction Mechanism of CCM−CMC

**Figure 3:**
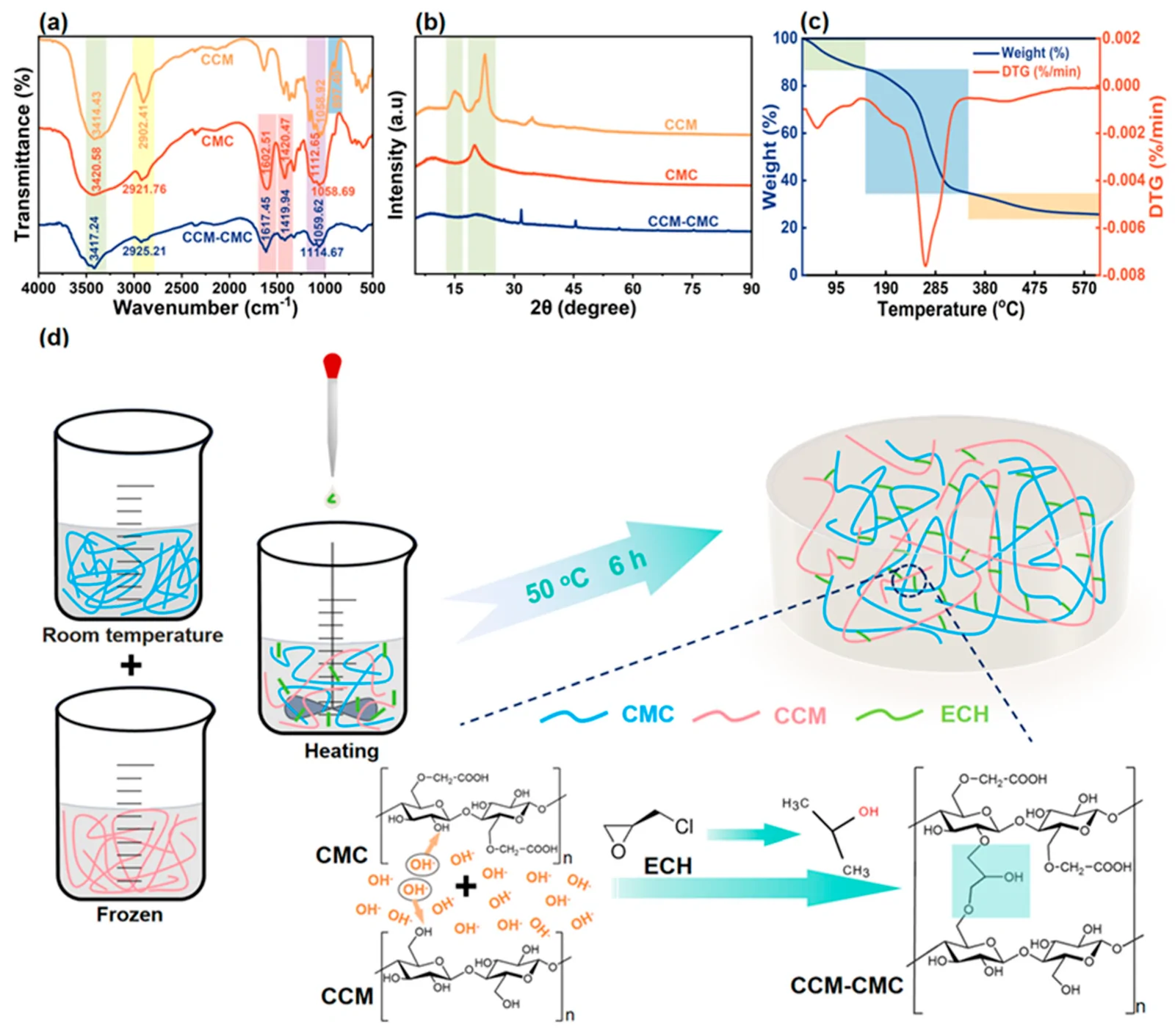
(**a**) FTIR and (**b**) XRD spectra of CCM, CMC, and CCM−CMC. (**c**) TGA of CCM−CMC. Schematic diagram of the (**d**) CCM−CMC reaction process.

Figure 3a presents the FTIR spectra of CCM, CMC, and CCM−CMC. In this figure, the absorption band observed at 897.40 cm^−1^ is assigned to the β-glycosidic linkage, a feature that is characteristic of cellulose. CMC exhibits COO^−^ asymmetric stretching vibration peaks at 1602.51 cm^−1^ and COO^−^ symmetric stretching vibration peaks at 1420.47 cm^−1^. The band observed at 1058.69 cm^−1^ is attributed to the C–O stretching vibration originating from the cellulose backbone [7]. The CCM−CMC infrared spectrum simultaneously exhibits characteristic peaks of both CCM and CMC. The absorption peaks at 1059.62 cm^−1^ and 1114.67 cm^−1^ originate from C-O (C-O-C) stretching vibrations, stemming from the ether bonds formed by the crosslinking of the cellulose backbone and epichlorohydrin. These spectral features conclusively demonstrate the successful synthesis of the CCM−CMC material [20].

The XRD patterns of CCM, CMC, and CCM−CMC are presented in Figure 3b. It can be seen that CCM exhibits a distinct absorption peak at 2θ ≈ 15° and a strong, sharp diffraction peak at 2θ ≈ 22°, which are typical crystalline characteristics of cellulose. In the CMC pattern, the sharp characteristic peak of native cellulose (2θ ≈ 22°) exhibits significantly reduced intensity and broadened peak shape. This results from the carboxymethylation modification disrupting the regular structure of the crystalline regions in cellulose, leading to a substantial decrease in crystallinity and a shift toward an amorphous state. In the CCM−CMC pattern, the strong crystalline peak characteristic of CCM is almost entirely absent, replaced by a broadened signal of low intensity. The overall diffraction intensity is substantially lower than that of both CCM and CMC. This indicates that during gel preparation, the crystalline structure of CCM was further disrupted. Additionally, the entanglement of CCM and CMC molecular chains, coupled with hydrogen bonding interactions, suppressed crystallization within the system. Ultimately, this led to the formation of a polymer network dominated by an amorphous structure.

The thermal weight loss curve and weight loss rate curve of CCM−CMC are illustrated in Figure 3c. The first stage, occurring between 30 °C and 150 °C, represents the evaporation of adsorbed water and free water within the WRAs. The 150–350 °C range represents the primary weight loss phase for the WRAs, corresponding to the thermal depolymerization of cellulose molecular chains, glycosidic bond cleavage, and polymer degradation. This constitutes the typical main degradation zone for cellulose-based materials. A distinct peak in the weight loss rate observed during this phase indicates that this temperature range corresponds to the most intense degradation reaction. The hydrogen bonds between CCM and CMC build a cross-linked secondary network to limit molecular chain motion and improve the thermal stability of the prepared water-retaining agent. Dang et al. [33] reported that incorporating CMC into polyacrylamide hydrogels constructed a second network via hydrogen bonding, leading to enhanced thermal stability of the hydrogels. Beyond 350 °C, the decline in the thermogravimetric curve significantly slows, eventually stabilizing at approximately 25%. This corresponds to the slow thermal decomposition and volatilization of the residual char following degradation.

Based on the results in Figure 3a–c, the cross-linking reaction mechanism of CCM and CMC is proposed, as depicted in Figure 3d. First, CMC and CCM undergo activation in a sodium hydroxide alkaline environment. Hydroxide ions in the solution attack the hydrogen atoms in the hydroxyl groups of the cellulose molecular chains, converting the hydroxyl groups into more nucleophilic oxygen anions (O^−^) [20]. Subsequently, the epoxy group in ECH molecules undergoes ring-opening in the alkaline environment. The oxygen anions generated on the CCM and CMC molecular chains act as nucleophiles, attacking the carbon atom with lower electron density in the epoxy bond. This promotes the ring-opening reaction of the epoxy bond, forming an intermediate containing a chlorinated alcohol structure. Finally, the reactive group on the opened intermediate reacts with the hydroxyl group (or activated oxygen anion) on another CCM or CMC molecule, eliminating a chloride ion or water molecule. This ultimately forms a stable ether-bonded crosslinked structure, linking different molecular chains to construct a three-dimensional crosslinked network.

### 3.3. Analysis of the Swelling Properties of CCM−CMC

**Figure 4:**
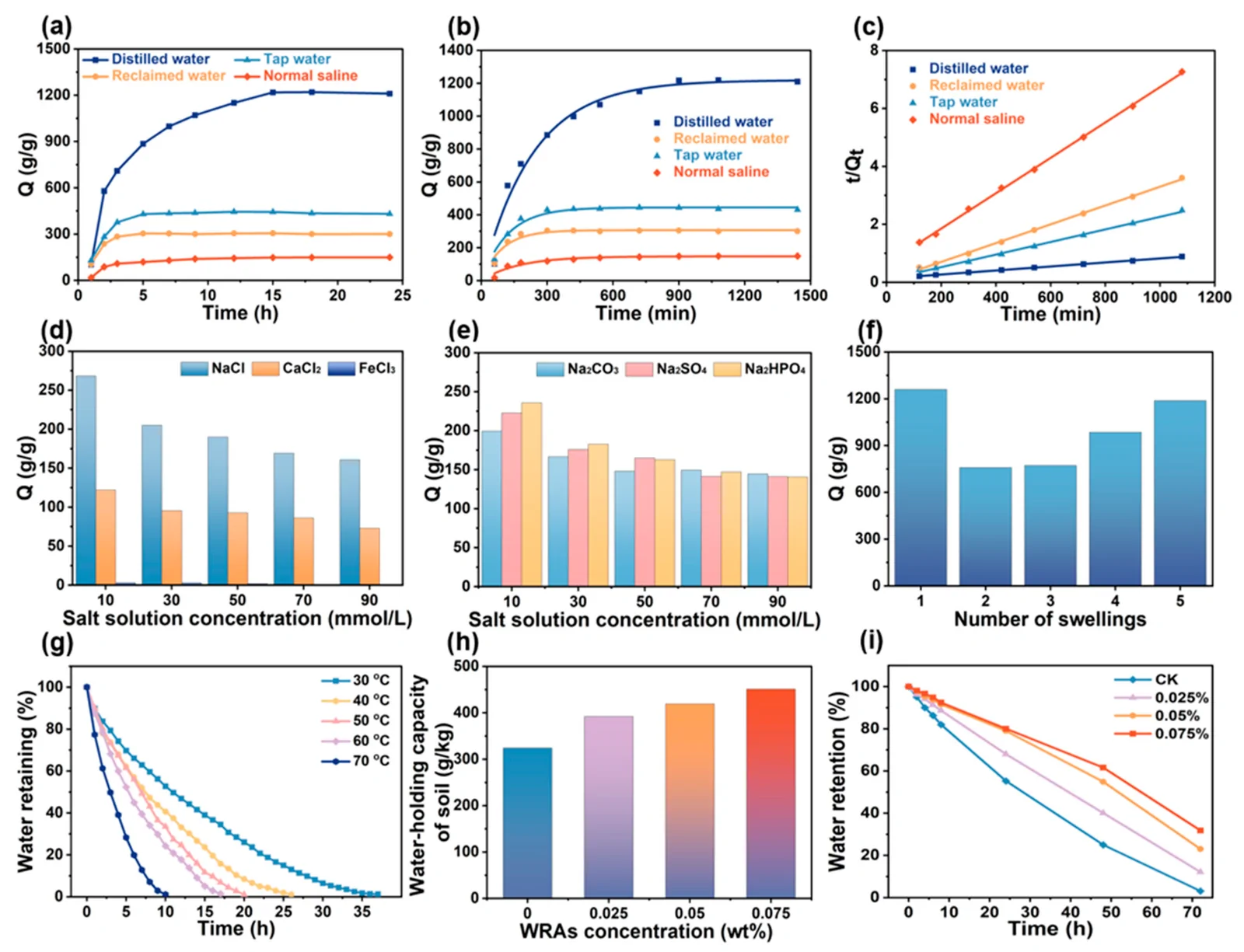
CCM−CMC Swelling Performance Testing. (**a**) Swelling tests in different liquids. (**b**) Pseudo-first-order kinetic fitting of the swelling process. (**c**) Pseudo-second-order kinetic fitting of the swelling process. (**d**,**e**) Swelling performance in different salt solutions. (**f**) Cyclic swelling performance. (**g**) Water retention capacity at different temperatures. (**h**) Effect of CCM−CMC dosage on soil water-holding capacity. (**i**) Effect of CCM−CMC dosage on soil evaporation rate.

Figure 4a presents the swelling rate curves of CCM−CMC in distilled water, reclaimed water, tap water, and physiological saline (0.9 wt% NaCl). It can be seen from the figure that CCM−CMC achieves the highest swelling ratio in distilled water, reaching swelling equilibrium at approximately 15 h with an equilibrium swelling ratio of 1217.84 g/g, whereas the equilibrium swelling ratio in physiological saline is the lowest (147.42 g/g). This is because the ions, organic substances and other components contained in reclaimed water, tap water and physiological saline not only disrupt the hydrogen bonding interactions within the three-dimensional network of CCM−CMC, but also elevate the ionic osmotic pressure of the solution and enlarge the osmotic pressure difference between the inside and outside of the CCM−CMC network, thereby inhibiting the water absorption and swelling process [8]. To further explore the swelling process of CCM−CMC, the pseudo-first-order and pseudo-second-order kinetic models were adopted to fit the swelling process, and the fitting results are shown in Figure 4b,c.

According to the fitting data presented in Table 1, the R^2^ value for the pseudo-second-order kinetic model is considerably greater than that for the pseudo-first-order model. This indicates that the pseudo-second-order model provides a better description of the swelling kinetics of this water-retaining agent. In terms of the rate constant *k*, CCM−CMC swells the fastest in reclaimed water, while the swelling rate is relatively slow in distilled water and physiological saline. The high ionic strength of physiological saline compresses the gel network via the salting-out effect, which reduces the swelling rate; in contrast, distilled water has a simple composition, leading to a relatively low swelling driving force (osmotic pressure) and thus the slowest swelling rate. The fitting data lead to the conclusion that the pseudo-second-order kinetic model better describes the swelling behavior of CCM−CMC across various solutions; the findings in this work are similar to the conclusions reported by Wu et al. [20].

To clarify the effects of metal ions and anions on the swelling performance of CCM−CMC, the swelling properties of CCM−CMC were measured in solutions containing metal ions with different valences and anions of different species, as shown in Figure 4d,e. As illustrated in Figure 4d, the swelling ratio of CCM−CMC dropped considerably as the metal ion concentration increased. Moreover, at the same concentration, a higher valence of metal ions also led to a notable reduction in the swelling ratio of the water-retaining agents. The underlying reason is that the elevated ion concentration directly increases the ionic strength of the solution, which compresses the hydrophilic network of the water-retaining agents. In addition, high-valence metal ions can provide a higher ionic strength at the same concentration, and their electrostatic interaction with the negatively charged groups in the water-retaining agents network is stronger, resulting in a more remarkable shrinkage effect on the network structure and thus a more prominent inhibitory effect on swelling [34]. Figure 4e shows the variations in water absorption capacity of CCM−CMC in sodium salt solutions with different anions, and its swelling performance decreased with the increase in solution concentration. At the same concentration, the swelling ratios of CCM−CMC in different anion solutions were extremely close, indicating that the type of anions exerted a negligible effect on the swelling ratio of the water-retaining agents. This is mainly because the prepared CCM−CMC network contains a large number of negatively charged hydrophilic groups, and cations with opposite charges have a significant impact on the hydrophilic groups in the cross-linked network. Therefore, metal ions have a more pronounced effect on the swelling ratio of the water-retaining agents than anions [35].

For water-retaining agents used in arid environments, the ability to repeatedly absorb water and swell is an essential performance metric. The swelling property of CCM−CMC after 5 repeated swelling cycles is shown in Figure 4f. The results indicate that the swelling performance of CCM−CMC exhibits a trend of first decreasing and then increasing with the number of repetitions. During the first repeated swelling, partial collapse of the porous structure occurs during the drying process of the water-retaining agents, leading to a decrease in the swelling ratio. With the increase in the number of repeated swelling cycles, the continuous infiltration of water molecules disrupts part of the cross-linked network, causing the originally dense structure to dissociate into fragments and the pore size distribution to increase; meanwhile, the hydrogen bonds within the cross-linked network gradually break, the network structure tends to loosen, and more hidden hydrophilic sites are exposed, ultimately promoting the recovery of the swelling ratio. This result confirms that CCM−CMC possesses excellent reusable stability and is a sustainable water-absorbing and water-retaining material.

Figure 4g–i displays the results of additional experiments conducted to evaluate the real-world applicability of CCM−CMC, specifically its high-temperature water retention and its effectiveness in enhancing soil moisture retention. CCM−CMC after swelling equilibrium completely lost water after 37 h at 30 °C; with the increase in temperature, the water loss rate accelerated, and it could still retain water for 10 h even at a high temperature of 70 °C. It can be seen from Figure 4h that the soil water-holding capacity increased significantly with the increase in CCM−CMC dosage. When the dosage of CCM−CMC was 0.075 wt%, the soil water-holding capacity increased by 39.20% compared with the control group. This study shows that WRA application increases the soil water content and LRWC compared with the non-WRA treatment, consistent with previous findings in wheat [36] and oats [37]. The results of the soil water evaporation simulation experiment shown in Figure 4i further indicate that the soil water evaporation rate decreased significantly with the increase in CCM−CMC dosage. The above results fully demonstrate that CCM−CMC can effectively improve soil water-holding capacity, delay water evaporation, and possess a certain degree of high-temperature tolerance. For regions that are arid or semi-arid, this offers important benefits in terms of water-saving agriculture and restoring damaged ecosystem.

### 3.4. Safety Analysis

The safety of biodegradable water-retaining agents is a core prerequisite for their practical application in scenarios such as soil improvement and mining area restoration. In this study, the safety of CCM−CMC was comprehensively evaluated through cytotoxicity assays, animal skin contact toxicity tests, and soil degradation tests. The CCK-8 assay was used to determine the damage of CCM−CMC extracts to cells, and the cell viability results are presented in Table 2. After 24 h of incubation with CCM−CMC extracts of different concentrations, the cell viability in all experimental groups was higher than that of the control group (100%). These results confirm that CCM−CMC is non-cytotoxic and possesses good biocompatibility.

The dermal contact safety of CCM−CMC was evaluated via a single acute sensitization test on mice. Through a single dermal exposure, the acute sensitization potential of the material (i.e., the risk of inducing allergic reactions such as skin erythema and edema) was determined. The results of skin reactions in mice from the acute dermal irritation test are presented in Table 3. The scoring criteria for the mouse acute dermal test (Tables S1 and S2) and the photographs of mouse tests (Figure S1) are available in the Supplementary Materials.

No erythema or edema was observed in the control group at 24 h, 48 h, and 72 h (mean score = 0). Only slight, barely perceptible erythema was found in the experimental group at 72 h (mean score = 0.333). According to the grading criteria for dermal irritation intensity, the experimental group was classified as non-irritating. The results of this test indicate that CCM−CMC poses no risk of acute dermal contact irritation to mice, providing certain safety evidence for its practical application.

**Figure 5:**
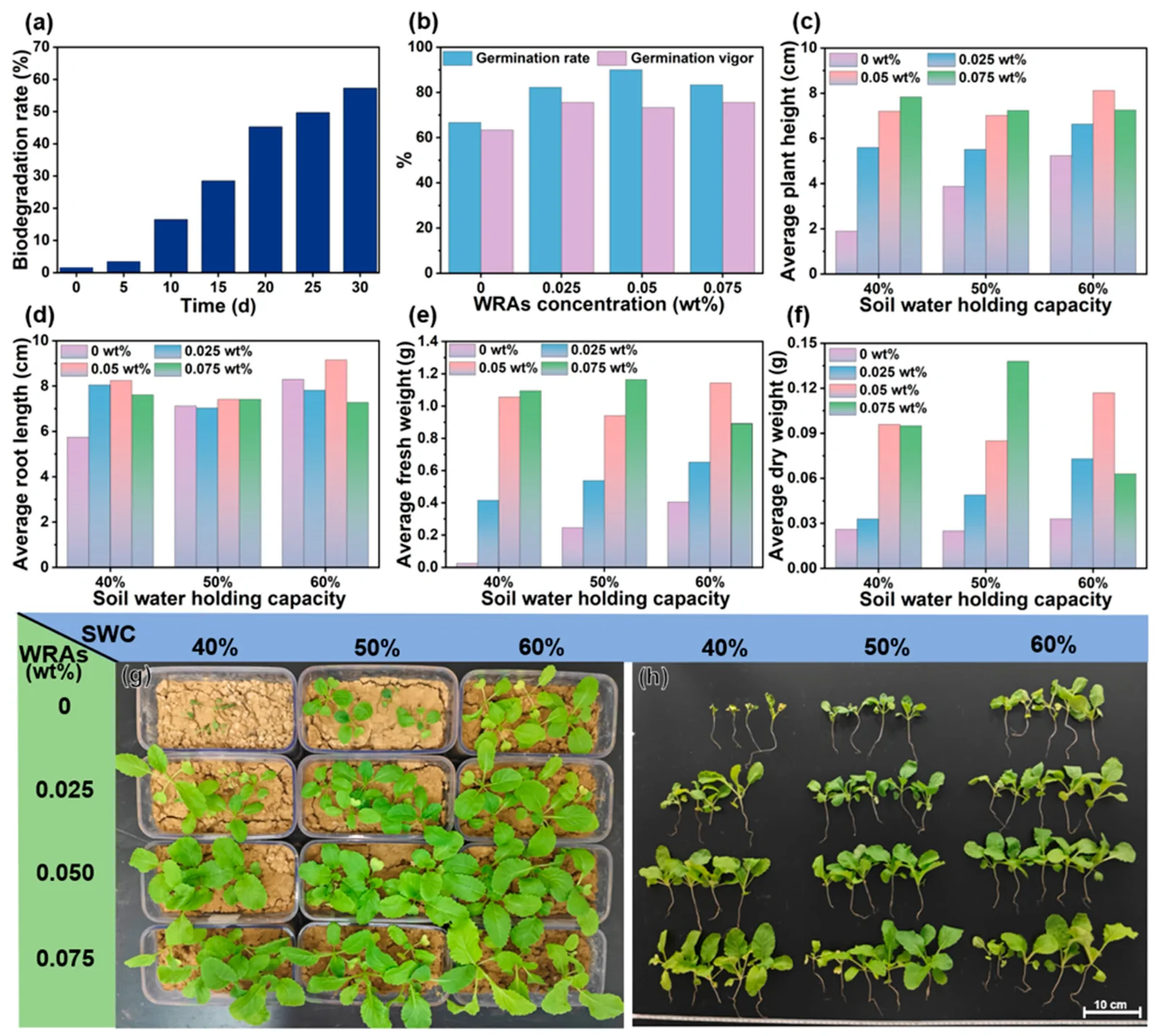
Soil biodegradation rates of CCM−CMC and its effects on growth indicators and planting performance of pakchoi under drought conditions at different application rates. (**a**) Soil biodegradation rates of CCM−CMC. (**b**) Effects of different CCM−CMC application rates on germination rate and vigor of pakchoi under drought gradients. (**c**–**f**) Average plant height, average root length, average fresh weight, and average dry weight of Chinese cabbage. (**g**,**h**) Comparative images of simulated pakchoi cultivation under drought conditions.

The soil biodegradation rate of CCM−CMC is shown in Figure 5a. Under arid mining soil conditions, the weight loss of CCM−CMC reaches approximately 57.28% within 30 d, with a half-life of around 25 d. This result confirms that CCM−CMC is readily biodegradable even in arid mining soils with low organic matter and high salinity-alkalinity [20].

### 3.5. Study on Simulation of Drought Stress Cultivation in Pakchoi and the Regulatory Effects of Water Retention Agents

By simulating the drought environment, different CCM−CMC dosage treatment groups and a blank control group were set up. The growth indicators and physiological characteristics of pakchoi were systematically monitored to evaluate the effect of CCM−CMC on the germination and growth of pakchoi under drought stress. The planting results of pakchoi are shown in Figure 5b–h, and the mortality during the growth process is shown in Supplementary Materials, Table S3.

The germination and growth of pakchoi are sensitive to water; drought or excessive water can affect its growth. As can be seen from Figure 5b, the application of CCM−CMC facilitates the germination of pakchoi. Among them, the germination rate of pakchoi treated with 0.05 wt% CCM−CMC was the highest, reaching 90.0%, which was 23.33% higher than that of the blank control group. The germination energy was also improved by approximately 19.31%. The increase in germination rate proves that the water absorption and retention performance of CCM−CMC provides adequate water supply for the germination of pakchoi under drought conditions. A high germination energy indicates rapid and uniform seedling emergence, strong seedlings, excellent stress resistance, and a high seedling survival rate. After thinning, five pakchoi seedlings remained in each group. Some seedlings still died during subsequent growth, and the mortality data are presented in Table S3 in the Supplementary Materials.

Under the same drought conditions, the application of different amounts of CCM−CMC increased the plant height, root length, fresh weight, dry weight, and other indicators of pakchoi to varying degrees compared with the blank control group. The average plant height increased by a maximum of 327.4%, and the average dry weight increased by a maximum of 430.8%. At 40% soil water-holding capacity (severe drought), all pakchoi withered due to water shortage; however, under the same drought conditions, pakchoi grew well after adding CCM−CMC. At 60% soil water-holding capacity (mild drought), the growth performance of pakchoi treated with 0.075 wt% CCM−CMC was poorer than that treated with 0.05 wt% CCM−CMC. This is because a higher dosage of CCM−CMC can absorb and retain more water, thereby affecting soil aeration and further inhibiting the growth of pakchoi. Therefore, an appropriate dosage of CCM−CMC should be ensured in practical applications. It can be clearly seen from Figure 5g that the soil moisture content varies with the application of CCM−CMC, which visually confirms the excellent water absorption and retention performance of CCM−CMC [38].

### 3.6. Analysis of Field Experimental Results for Mine Ecological Restoration

The arid mining area soil in Xinjiang is characterized by prominent problems such as strong saline-alkali properties, extremely low organic matter content, and a soil matrix dominated by hard mudstone, resulting in difficulties in vegetation colonization and growth, with large areas of bare land in the region. To address the ecological remediation needs of such mining areas, the traditional soil replacement remediation model has drawbacks, including high cost and easy introduction of exogenous substances that disturb the local native ecology. In addition, the mining area ecosystem has a fragile background and an extremely low tolerance threshold to external disturbances. Therefore, in situ remediation of native soil that avoids the input of exogenous substances has become the preferred technical approach for ecological governance in this region. Previous studies have demonstrated that the application of water-retaining agents can effectively alleviate soil salinity-alkalinity stress, improve soil quality and promote plant growth [31,32].

**Figure 6:**
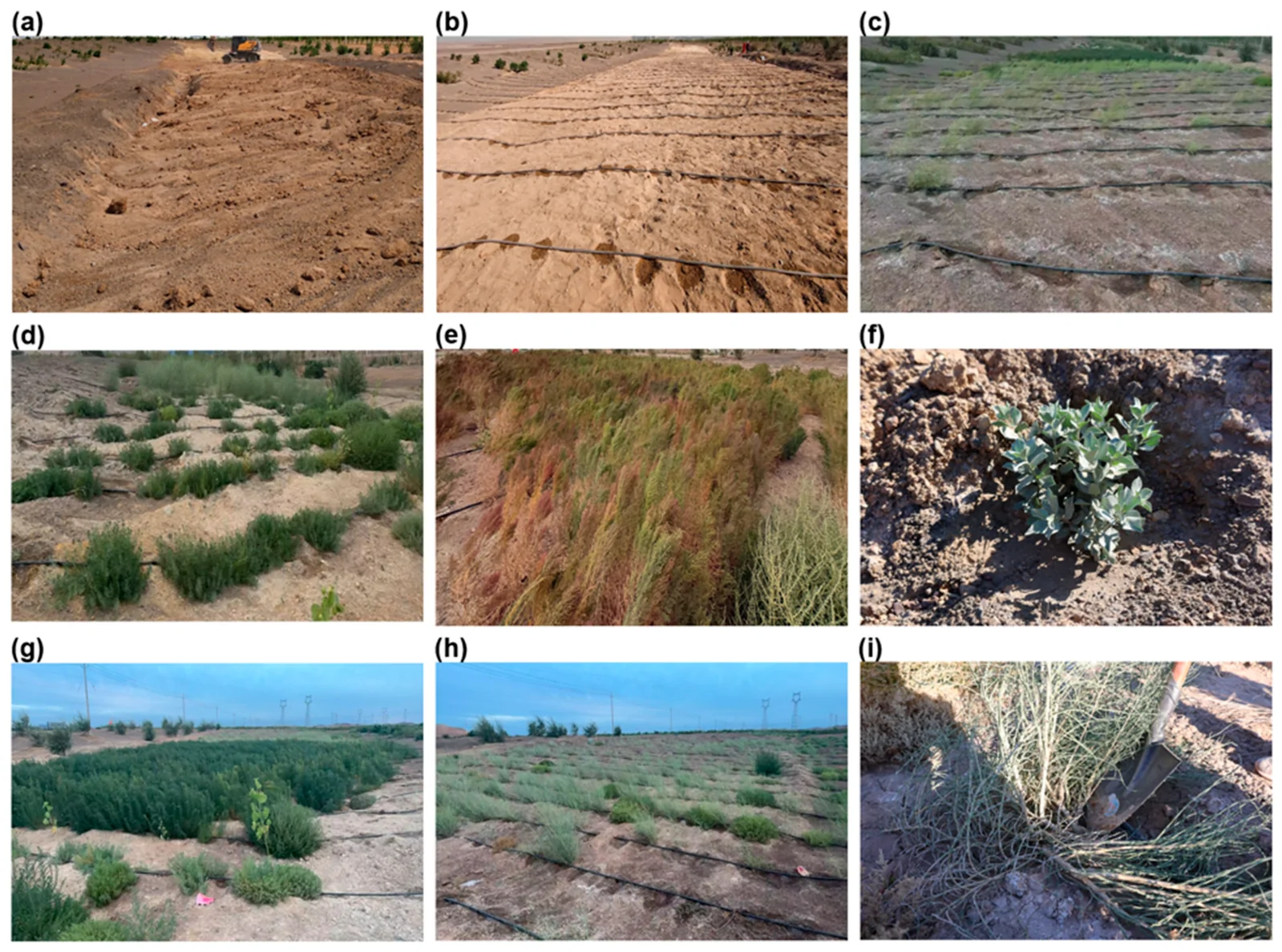
Remediation System Combining CCM−CMC with Biofertilizer Application. (**a**) Land leveling. (**b**) Seeding. (**c**) Plant growth at 30 days. (**d**) Plant growth at 60 days. (**e**) Plant growth at 90 days. (**f**) *Ammopiptanthus mongolicus*. (**g**) *Suaeda glauca*. (**h**) *Hedysarum scoparium*. (**i**) *Sacsaoul*.

This study constructed a remediation system based on the synergistic application of CCM−CMC WRA and biological fertilizer. Native plants with cold-resistant, drought-tolerant, and salt-tolerant properties, such as *Suaeda glauca*, *Ammopiptanthus mongolicus*, *Sacsaoul*, and *Hedysarum scoparium*, were selected for the remediation of saline-alkali and barren soil in mining areas. The planting results are shown in Figure 6. To meet the high demand of CCM−CMC water-retaining agent, scale-up experiments were conducted and a kilogram-scale production setup was established, with related data included in the Supplementary Materials (Figure S2 and Table S4). It can be seen from the figure that in the test area improved by CCM−CMC, the overall survival rate of plants exceeded 80%. The plants grew well with well-developed root systems, and the mudstone properties of the soil in the planting area gradually diminished, with the soil structure significantly improved. A systematic comparison of the physicochemical properties and fertility parameters of the habitat soil versus the planted soil is provided in Table 4. The results indicate that the original habitat soil was a typical salinized soil, characterized by an extremely low organic matter content, a high pH value, and a total salt content as high as 20 g/kg. Applying CCM−CMC together with plants adapted to salty and dry conditions led to significant positive shifts in soil physicochemical properties. Fertility indicators (organic matter, NO_3_^−^-N, NH_4_^+^-N, available P, available K) all increased, while soil pH declined and the total salt content was greatly lowered. Among them, the soil salt reduction rate in the *Suaeda* glauca planting plot was the most prominent, highlighting the unique advantage of this species in the desalination and improvement of severe saline-alkali soil. In contrast, *Hedysarum scoparium* exhibited excellent remediation effects and broad prospects for popularization and application due to its characteristics of low water requirement and strong environmental adaptability.

Long-term desert restoration is a gradual process. The required application times of amendments depend on local climate, soil salinity, rainfall and plant survival status. Generally, multiple fertilizer topdressing events within the five-year period may be needed to maintain soil nutrient supply, together with continuous monitoring and maintenance, so as to achieve stable vegetated land. Regrettably, no viable germination and growth occurred in the control treatments with separate applications of CCM−CMC water-retaining agent, biofertilizer and native plants. Additional studies are needed to fully elucidate the synergistic effects of these three components. For example, inspired by Zhang et al., the EWM–TOPSIS–AISM model can be used to integrate multiple objectives and identify the optimal management practice [39].

## 4. Conclusions

To address the ecological restoration challenges of arid, frigid, saline-alkali mining areas, this study discarded the conventional soil replacement remediation approach. A cellulose-based biodegradable water-retaining agent (CCM−CMC) was developed, and an in situ non-soil-repair restoration system combining water-retaining agent, biofertilizer and native stress-tolerant plants was constructed to realize efficient soil improvement and vegetation reconstruction.

CCM−CMC exhibits excellent water absorption/retention performance and biodegradability. After soil incorporation, soil water-holding capacity increased by up to 39.20%, and the material had a weight loss of 57.28% following 30 days of soil burial. Without exogenous replacement soil, the agent can effectively improve the in situ soil structure of mining areas, loosen mudstone, raise organic matter and available nutrients, and reduce soil pH and total salt content.

Field trials show that the survival rate of native plants (*Haloxylon ammodendron* and *Suaeda glauca*) exceeds 80%. This study provides a referable technical route for the ecological restoration of arid and heavily saline-alkali mining areas. The planted plants in the mining area are thriving to date; our field monitoring is still in progress, and the long-term restoration performance has been preliminarily confirmed. Nevertheless, substantial challenges remain for its large-scale practical application, especially in scaling up production and reducing manufacturing costs for widespread adoption. Long-term monitoring and research are still required to investigate whether chemical residues are generated during CCM−CMC degradation and their associated environmental impacts. In addition, more extensive and in-depth studies are needed to fully elucidate the persistence of hydrogels in various soil types, as well as their complex interactions with soil physicochemical properties and nutrient dynamics.

## Figures and Tables

**Table 1:** Data of fitting parameters for the CCM−CMC swelling kinetics.

Swelling Medium	Pseudo-First-Order Kinetic		Pseudo-Second-Order Kinetic	
	k_1_/min^−1^	R^2^	k_2_·10^−4^/g·g^−1^·min^−1^	R^2^
Distilled water	0.00429	0.95851	0.00038	0.99911
Reclaimed water	0.01060	0.92115	1.99764	0.99870
Tap water	0.00839	0.95447	0.54463	0.99616
Normal saline	0.00586	0.92091	0.59017	0.99929

**Table 2:** Results of CCK-8 cytotoxicity assay.

Group	Control	50	25	12.5	6.25	3.13	Blank
		mg/mL	mg/mL	mg/mL	mg/mL	mg/mL	
A	2.5638	2.6039	2.6793	2.5184	2.4179	2.6845	0.1511
B	2.5889	2.6825	2.6479	2.7453	2.6581	2.5857	0.1442
C	2.6245	2.5883	2.6261	2.587	2.9079	2.5979	0.1276
Average	2.5924	2.6249	2.6511	2.6169	2.6613	2.6227	0.1410
Correct	2.4514	2.4839	2.5101	2.4759	2.5203	2.4817	--
Cell viability (%)	100	101.325	102.395	100.999	102.811	101.236	--

**Table 3:** Results of acute dermal irritation test on mouse skin.

Group	Average Score				Highest Score	Stimulus Intensity	Mortality Rate (%) (*n* = 3)
	d0	d1	d2	d3			
Control	0	0	0	0	0	Non-irritating	0
Experimental	0	0	0	0.333	0.333	Non-irritating	0

**Table 4:** Analysis of the physicochemical properties and fertility index evaluation of habitat soil and cultivation soil.

Sample Name	Organic Matter	NO_3_-N	NH_4_-N	Quick-Acting Phosphorus	Fast-Acting Potassium	pH	Salt Content
	g/kg	mg/kg	mg/kg	mg/kg	mg/kg		g/kg
*Habitat soil*	3.456 ± 0.24	29.147 ± 0.83	5.231 ± 0.21	2.168 ± 0.09	93.740 ± 2.10	7.82 ± 0.06	17.075 ± 0.41
*Ammopiptanthus mongolicus* planting soil	5.424 ± 0.18	72.654 ± 1.12	6.111 ± 0.24	3.161 ± 0.12	180.105 ± 1.87	7.23 ± 0.11	12.065 ± 0.29
*Suaeda glauca* planting soil	7.317 ± 0.15	82.570 ± 0.96	10.561 ± 0.32	3.365 ± 0.10	269.492 ± 0.93	7.19 ± 0.10	9.740 ± 1.02
*Hedysarum scoparium* planting soil	6.769 ± 0.20	84.563 ± 1.03	8.538 ± 0.26	2.810 ± 0.09	217.976 ± 2.24	7.21 ± 0.04	11.440 ± 0.97
*Sacsaoul* planting soil	5.813 ± 0.12	63.719 ± 0.86	8.136 ± 0.13	2.489 ± 0.14	226.627 ± 1.56	7.47 ± 0.13	11.240 ± 1.09

All data are expressed as mean ± standard deviation (SD) of three parallel replicates.

## Data Availability

The raw data are not publicly available due to privacy restrictions, but the analyzed data that support the findings of this study are available from the corresponding author upon reasonable request.

## Supplementary Materials

The following supporting information can be downloaded at: https://www.mdpi.com/article/10.3390/polym18212670/s1, Figure S1: Photograph of mouse skin in acute skin irritation test, Figure S2: (a) Kilogram-scale production facility. (b) Digital photograph of the kilogram-scale product; Figure S3: SEM micrographs of CCM-CMC. (a,b) First swelling cycle. (c,d) Fifth swelling cycle; Table S1: Skin irritation response rating scale, Table S2: Skin irritation severity grading scale, Table S3: Mortality of pakchoi seedlings during drought simulation (*n* = 5), Table S4: Comparison of kilogram-scale and laboratory-scale products. File S1: Author Checklist (ARRIVE).
