# Supplementary material for: A Sustainable Approach: Cellulose-Based Biodegradable Water-Retaining Agents Facilitating Mine Restoration in Arid Xinjiang Mining Zones
Source: Polymers (Basel). 2026 Oct 28;18(21):2670. doi: 10.3390/polym18212670 (PMC13766150; doi:10.3390/polym18212670)
Supplement: Supplementary file 1 [file polymers-18-02670-s001.zip › polymers-4605115-supplementary resub.pdf]

A sustainable approach: cellulose-based biodegradable water-retaining agents facilitating mine restoration in arid Xinjiang mining zones

Kaigang Li [a,b], Naeem Akram [c,*], Lei Zhou [d], Zhanwen Cao [b], Huishan Zhang [b], Ali Ahmad [c,e], Zhenning Cheng [b], Aizong Wu [a], Wenlong Mo [a], Jia Guo [b,*]

[a] School of Chemical Engineering, Xinjiang University, Urumqi 830046, Xinjiang, China

[b] Xinjiang Energy Co, LTD, Urumqi 830000, Xinjiang, China

[c] School of Chemical Engineering, Minhaj University Lahore, Lahore 54000, Pakistan

[d] Administration Committee of Chemical Industry Park, Midong District, Urumqi 830000, Xinjiang, China

[e] School of Mechanical Engineering, Shandong University of Technology, Zibo 255000, China

*Corresponding authors E-mail: naeemakram63@gmail.com (Naeem Akram), gj459824057@126.com (Jia Guo)

Table S1 Skin irritation response rating scale

Skin reaction	Points
**Erythema and eschar formation**	
No erythema	0
Mild erythema (barely visible)	1
Prominent erythema	2
Moderate to severe erythema	3
Severe erythema (purplish-red) with slight eschar formation	4
**Edema formation**	
No edema	0
Mild edema (barely visible)	1
Mild edema (skin swelling with distinct contours)	2
Moderate edema (skin elevation approximately 1 mm)	3
Severe edema(skin indentation exceeding 1 mm, with increased area)	4
Highest score	8

Table S2 Skin irritation severity grading scale

Mean score	Intensity
0~0.4	Non-irritating
0.5~1.9	Mildly irritating
2.0~5.9	Moderately irritating
6.0~8.0	Strongly irritating

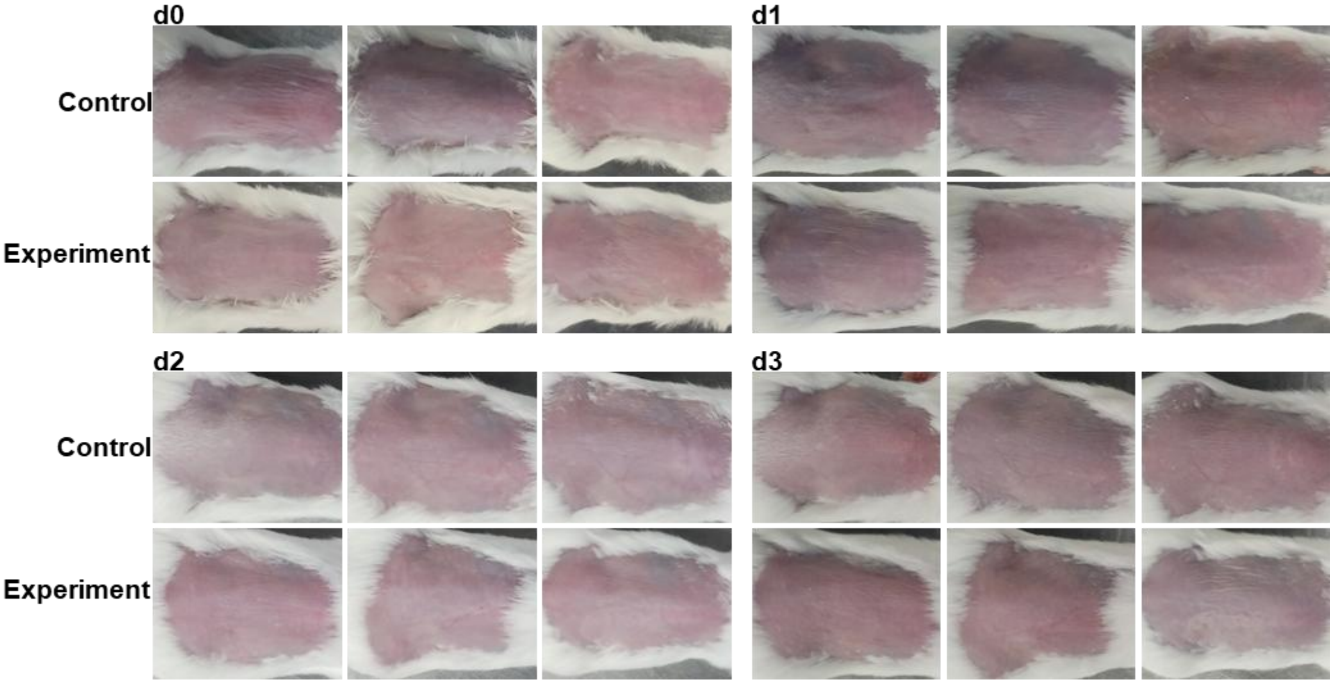

Fig. S1 Photograph of mouse skin in acute skin irritation test

Table S3 Mortality of pakchoi seedlings during drought simulation (n=5)

WRAs content	Soil water-holding capacity		
	40%	50%	60%
0 wt%	5	1	0
0.025 wt%	1	0	0
0.05 wt%	1	0	0
0.075 wt%	0	0	0

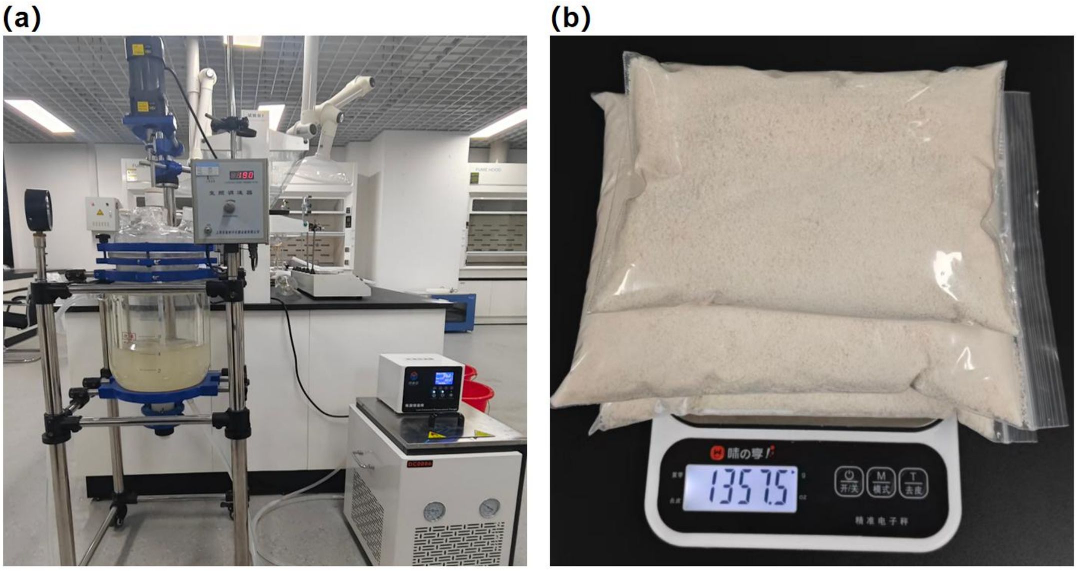

Fig. S2 (a) Kilogram-scale production facility. (b) Digital photograph of the kilogram-scale product.

Table S4 Comparison of kilogram-scale and laboratory-scale products

Products	Swelling ratio (g/g)			
	distilled water	tap water	normal saline	reclaimed water
laboratory-scale	1217.84	443.09	148.1	304.96
kilogram-scale	1041.18	386.82	131.07	270.50

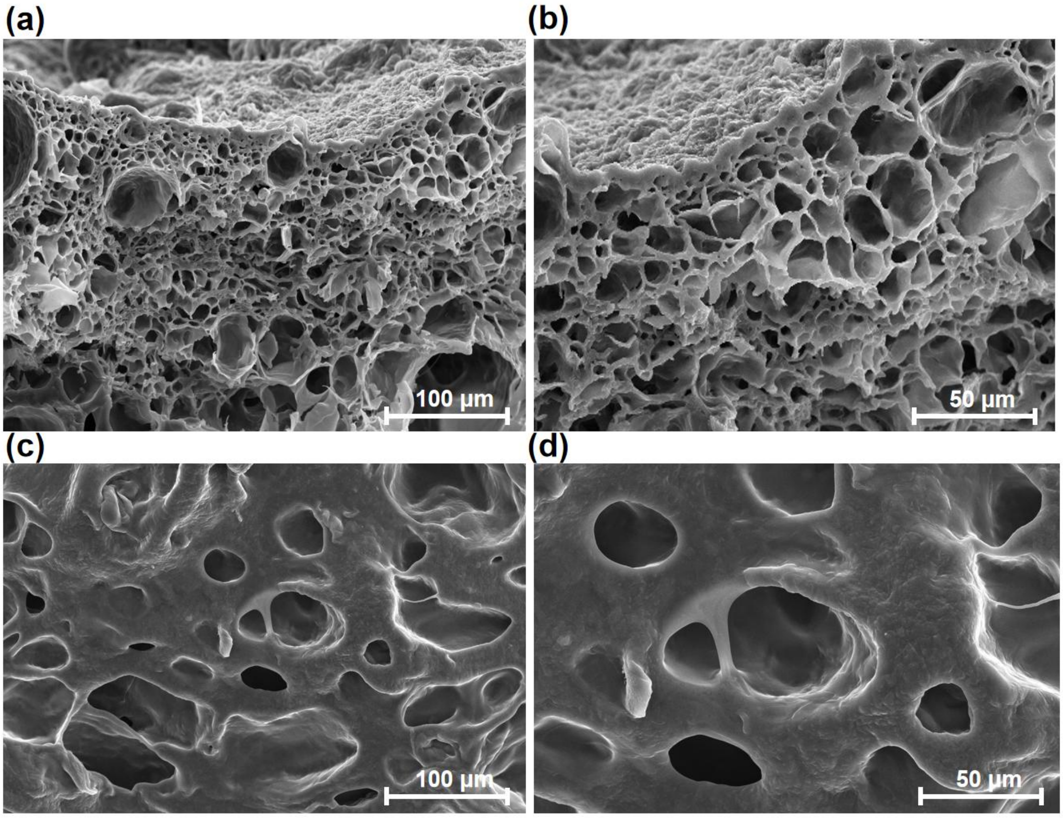

Fig. S3 SEM micrographs of CCM-CMC. (a,b) First swelling cycle. (c,d) Fifth swelling cycle.
